# Coactivators in PPAR-Regulated Gene Expression

**DOI:** 10.1155/2010/250126

**Published:** 2010-08-05

**Authors:** Navin Viswakarma, Yuzhi Jia, Liang Bai, Aurore Vluggens, Jayme Borensztajn, Jianming Xu, Janardan K. Reddy

**Affiliations:** ^1^Department of Pathology, Feinberg School of Medicine, Northwestern University, Chicago, IL 60611, USA; ^2^Department of Molecular and Cellular Biology, Baylor College of Medicine, Houston, TX 77030, USA

## Abstract

Peroxisome proliferator-activated receptor (PPAR)*α*, *β* (also known as *δ*), and *γ* function as sensors for fatty acids and fatty acid derivatives and control important metabolic pathways involved in the maintenance of energy balance. PPARs also regulate other diverse biological processes such as development, differentiation, inflammation, and neoplasia. In the nucleus, PPARs exist as heterodimers with retinoid X receptor-*α* bound to DNA with corepressor molecules. Upon ligand activation, PPARs undergo conformational changes that facilitate the dissociation of corepressor molecules and invoke a spatiotemporally orchestrated recruitment of transcription cofactors including coactivators and coactivator-associated proteins. While a given nuclear receptor regulates the expression of a prescribed set of target genes, coactivators are likely to influence the functioning of many regulators and thus affect the transcription of many genes. Evidence suggests that some of the coactivators such as PPAR-binding protein (PBP/PPARBP), thyroid hormone receptor-associated protein 220 (TRAP220), and mediator complex subunit 1 (MED1) may exert a broader influence on the functions of several nuclear receptors and their target genes. Investigations into the role of coactivators in the function of PPARs should strengthen our understanding of the complexities of metabolic diseases associated with energy metabolism.

## 1. Introduction

The foundation for the discovery and designation of the PPAR subfamily of nuclear receptors in the early 1990s is the cumulative work over the preceding 25 years with peroxisome proliferators, a group of structurally diverse chemicals that induce characteristic and predictable pleiotropic responses including the transcriptional activation of genes involved in the fatty acid oxidation [1–6]. The PPAR subfamily consists of three members namely PPAR*α* (NR1C1), PPAR*β* (also known as *δ*) (NR1C2), and PPAR*γ* (NR1C3) with a high degree of sequence conservation across the species [1, 2, 7–9]. All three PPARs in the human and mouse are encoded by separate genes that are on different chromosomes [9]. PPAR*γ* has two isoforms, PPAR*γ*1, and an N-terminal 30 amino acid extended form PPAR*γ*2, both encoded by the same gene using two distinct promoters and alternate splicing [10, 11]. All three members of PPAR subfamily function as sensors for fatty acids and fatty acid derivatives and control metabolic pathways involved in energy homeostasis [12, 13]. PPARs display high levels of homologies at the protein level, but exhibit distinct and noninterchangeable functional roles in mammalian energy metabolism [9]. PPAR*α* is expressed in tissues with high fatty acid oxidation activities, which include liver, kidney, small intestine, heart, and skeletal muscle, consistent with its predominant functional role in regulating lipid catabolism. In the liver, PPAR*α* is the master regulator of mitochondrial, peroxisomal, and microsomal fatty acid oxidation systems where it is activated by synthetic peroxisome proliferators and in addition senses the influx of fatty acids during fasting to upregulate the fatty acid burning capacity [14]. PPAR*α* also plays a role in lipoprotein synthesis, inflammatory responses and the development of cancer in the rodent liver [15–19]. PPAR*β* is ubiquitously expressed with relatively higher levels found in brain, adipose tissue, and skin [20]. Activation of PPAR*β* also induces expression of genes required for fatty acid oxidation and energy dissipation in skeletal muscle and adipose tissue which in turn lead to improved lipid profiles and reduced adiposity [21]. In the liver, PPAR*β* can be activated by plasma free fatty acids influxed during fasting conditions [22]. PPAR*γ* which is expressed at a relatively high level in adipose tissue serves as an essential regulator for adipocyte differentiation and promotes lipid/energy storage in mature adipocytes by increasing the expression of several key genes in this pathway [23]. These two important functions of PPAR*γ*, namely adipogenesis and fat storage in adipocytes account for the insulin sensitizing effects of the anti-diabetic thiazolidinediones [24]. In summary, PPAR*α* and PPAR*β* participate in energy burning, whereas PPAR*γ* is critical in regulating adipocyte differentiation and energy storage by adipocytes [11, 25, 26]. 

## 2. Transcriptional Regulation of PPARs

**Figure 1:**
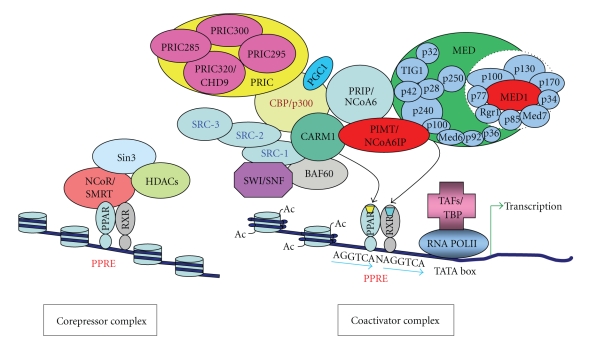
A schematic representation of ligand-dependent recruitment of coactivators for PPAR-regulated target gene transcription. In the absence of ligand, the PPAR-RXR heterodimer recruits corepressors, which in turn, assemble additional components of a repressor complex including histone deacetylase (HDAC). When ligand (yellow trapezium representing PPAR ligand, and blue trapezium representing 9-cis-retinoic acid as RXR ligand) binds, conformational changes in PPAR-RXR induce dissociation of corepressor complex. Active transcriptional complex assembles with coactivator proteins either sequentially or preassembled subcomplex modules. PPAR binds to peroxisome proliferator response element (PPRE) and assemble coactivator complexes that acetylate (SRCs, p300) or methylate (CARM1) nucleosomes for chromatin remodeling. Mediator components [36, 37] contact PPARs and facilitate the recruitment of the basal transcription machinery (TATA-box-binding protein [TBP]/TBP-associated factors [TAFs]) to form linkage with RNA polymerase II for transcription of specific target genes.

PPARs are ligand-activated transcription factors similar to other members of the nuclear hormone receptor superfamily [7, 8]. PPARs are nuclear in location, where they remain heterodimerized with the 9-cis retinoic acid receptor, RXR*α* (NR2B) [13] and bind to the upstream cis-acting regulatory regions termed as peroxisome proliferator response element (PPRE) of target genes [9, 27]. The canonical PPRE consists of two direct repeats AGGTCA separated by a single nucleotide so-called DR-1 element [28]. The two half-sites are distinguishable by their 5′ and 3′ positioning on the DR1 element whereby the DNA binding domain of PPAR binds 5′ half-site while RXR binds to the 3′ half-site [29, 30]. In addition to core DR-1 sequence, PPRE element contains an additional AAACT motif at the 5′ upstream region [30]. The hinge region of PPAR forms extensive interaction with the upstream AAACT element [30]. In the absence of ligand, the unliganded PPAR-RXR heterodimer remains bound to the nuclear receptor corepressor (NCoR) and silencing mediator of retinoid and thyroid hormone receptor (SMRT), two well-characterized corepressors (Figure 1) that are mostly present in the corepressor complex [31]. Both NCoR and SMRT directly interact with the Sin3 complex to form a multisubunit repressor complex [32]. SMRT functions as a platform protein and facilitates the recruitment of histone deacetylases (HDACs) to the DNA promoters bound by specific interacting transcription factors [32]. Another corepressor, Receptor Interacting Protein 140 (RIP140) also known as NRIP1 (nuclear receptor interacting protein 1) directly recruits HDAC and represses the activity of various nuclear receptor including PPARs by competing with their coactivators [33–35]. In the absence of ligand activation of nuclear receptor, the corepressor protein complex is known to subdue target gene transcription by causing the deacetylation of histones [31]. 

 The nuclear receptor-regulated transcriptional activation of target genes depends on the binding of a cognate ligand to the receptor (activator). For example, activation of PPAR*α*-RXR heterodimer by a PPAR*α* ligand triggers conformational changes in the receptor which releases the corepressor complex and recruits cofactor complexes to the promoter region of target genes (Figure 1) to initiate transcription [38, 39]. Transcription coactivators increase gene transcription via the acetylation of histones and through the recruitment and stabilization of the transcriptional complexes, mainly the Mediator complex which interacts directly with activator proteins and pol II [40–42]. During the past 15 years, more than 300 cofactors (coactivators, coregulators, corepressors etc.) have been identified but the in vivo physiological regulatory functions of many of these molecules in receptor/gene/cell-specific transcription remain to be explored [43]. This paper summarizes the current state of knowledge about the roles of coactivators and coactivator associated proteins (Table 1), with special emphasis on p160/SRC family members and PPAR-binding protein (PBP/PPARBP)/thyroid hormone receptor-associated protein 220 (TRAP220)/mediator complex subunit 1 (Med1), in the functioning of PPARs. 

## 3. Coactivators for PPAR Function

Transcriptional activation of PPAR-regulated genes is enhanced by coactivators. Most coactivators possess one or more LXXLL motifs (L: leucine and X: any amino acid) some of which may make contact with a coactivator-binding groove in the ligand-binding domain of nuclear receptor [44]. The assembly or preassembled coactivator complexes facilitate the liganded PPAR to achieve transcriptional activation of specific target genes in a tissue/cell-specific manner [45, 46]. Once coactivators are recruited to a liganded nuclear receptor they remodel chromatin structure by the intrinsic histone acetyltransferase (HAT) or methyl transferase activities [46]. In order to achieve this, steroid receptor coactivator (p160/SRC) family of proteins, which possess HAT activity, are recruited to the activation function 2 (AF-2) domain of the nuclear receptor and complex with the universal coactivator cyclic-AMP responsive element binding protein (CREB)-binding protein (CBP) and its homologue p300 [47–49]. CBP and p300 also exhibit potent HAT activity [50]. 

The second category of coactivators, with no known enzymatic functions, participates in the formation of a well-known multisubunit protein complex, variously called TRAP/DRIP/ARC/Mediator complex, consisting of 15–30 proteins [36, 37, 41, 57, 73–77]. Mediator complex, which is anchored by PBP (PPARBP)/TRAP220/DRIP205/Med1 facilitates interaction with poll II of the basal transcription machinery [41, 73, 75]. Members of this Mediator complex appear to be devoid of intrinsic enzymatic activities [46], but play an important role in connecting CBP/p300 bound coactivators with pol II containing preinitation complex [76]. Disruption of CBP/p300 and Med1 genes in the mouse results in embryonic lethality around E11.5 days, indicating that deletion of these pivotal anchoring coactivators affects the integrity of the cofactor complexes, thus altering the function of many nuclear receptors and most likely of other transcription factors [77–79]. 

A number of other coactivators and coactivator-associated proteins that possess enzymatic activities like methyltransferase (CARM1) [71], helicase (PRIC285, p68) [63, 80], and ATP dependent chromatin remodeling properties (PRIC320, SWI/SNF) [63, 80] or those without any enzymatic activities such as PPAR*γ* coactivator-1*α* (PGC-1*α*), PGC-1*β*, and BAFs [81, 82] have been identified in the active PPAR transcriptional complex, referred to as PRIC (PPAR*α*-receptor interacting cofactor) complex [64, 80]. Some other important coactivators such as PRIP (peroxisome proliferator-activated receptor-interacting protein)/ASC2/AIB3/RAP250/NCoA6 [61, 83–85] and PIMT (PRIP-interacting protein with methyltransferase domain/NCoA6IP) [70] are also identified, which serve as linkers between the initial HAT complex of CBP/p300 and p160 coactivators and the downstream mediator complex [76]. CCPG (constitutive coactivator of PPAR*γ*) is identified as a novel coactivator for PPAR*γ* [86]. As mentioned above, sequential or combinatorial recruitment of various multisubunit coactivator proteins on the liganded nuclear receptor leads to the establishment of a stable preinitiation complex with multiple possible configurations on to the target gene promoter. 

## 4. p160/SRC Family of Coactivators with HAT Activity

p160/SRC family of coactivators consists of three members, namely, SRC-1/NCoA-1, SRC-2/TIF2 (transcriptional intermediary factor 2)/GRIP1 (glucocorticoid receptor interacting protein 1), and SRC-3/pCIP (CBP-interacting protein)/RAC3 (receptor-associated coactivator-3)/ACTR/AIB1 (amplified in breast cancer-1)/TRAM-1(thyroid hormone receptor activator molecule 1) [51–55]. These proteins are required for mediating the transcriptional function of nuclear receptors and other transcription factors in a ligand-dependent manner [87, 88]. All three p160/SRC family members contain bHLH and PAS domains, which are involved in protein-protein interactions. These coactivators also contain three LXXLL motifs, that mediate recognition of, and binding to AF-2 region of a variety of nuclear receptors [89]. They possess HAT activity and are part of the first multiprotein coactivator complex with CBP/p300 on DNA bound-liganded nuclear receptors and participate in the acetylation of histones and remodel chromatin structure to unravel DNA for transcription [76, 88]. SRC-1 interacts with many nuclear receptors including PPAR*γ* and PPAR*α*, and the X-ray crystal structure of SRC-1 and the liganded PPAR*γ* complex revealed that binding is between highly conserved glutamate and lysine residues in the PPAR*γ* ligand binding domain and the backbone atoms of the LXXLL helices of SRC-1 [29]. Protein-protein interactions between PPAR*α* and SRC-1 and SRC-3 have been documented and these interactions appears to be ligand independent [52, 54]. p160/SRC family members exhibit strong sequence homology and somewhat similar functions under in vitro transactivation conditions. But gene knockout mouse models have provided valuable insights into the in vivo functional properties of these molecules [75, 88]. These mouse models include SRC-1^−/−^, SRC-2^−/−^ and SRC-3^-/^ single gene disruptions and SRC-1^−/−^/SRC-2^−/−^ and SRC-1^−/−^/SRC-3^−/−^ double nulls [50, 90–95]. 

**Figure 2:**
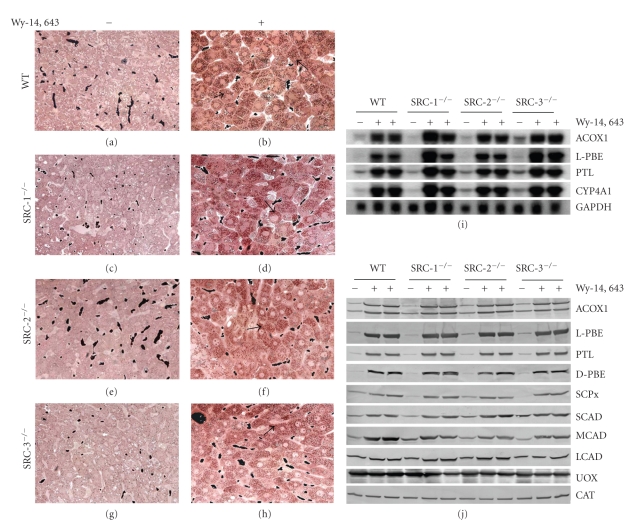
(a–h) Peroxisome proliferation in liver cells of wild-type (WT), SRC-1^−/−^ SRC-2^−/−^ and SRC-3^−/−^ mice treated with Wy-14,643 for 4 days. Liver sections were processed for the cytochemical localization of peroxisomal catalase by using alkaline 3′,3′-diaminobenzidine substrate. Control diet (upper panels; a, c, e, g). Wy-14,643 diet (lower panels; b, d, f, h). Peroxisomes appear as brown dots (arrows) distributed throughout the cytoplasm in these 0.5 *μ*m thick sections. All mice, wild-type and SRC nulls displayed extensive peroxisome proliferation after treatment with Wy-14, 643 indicating that these coactivators are not required for PPAR*α*-regulated pleiotropic responses including fatty acid oxidation. (i) Northern blot analysis to confirm changes in mRNA expression of peroxisomal and microsomal fatty acid metabolizing enzymes in wild-type and SRC nulls after 4-days treatment with PPAR*α* ligand Wy-14, 643. All genes are regulated by PPAR*α*. Fatty acyl-CoA oxidase-1 (ACOX1), peroxisomal enoyl-CoA hydratase/3-hydroxyacyl-CoA dehydrogenase bifunctional enzyme (L-PBE), and peroxisomal thiolase (PTL) represent peroxisomal *β*-oxidation system while CYP4A1 is involved in microsomal *ω*-oxidation of fatty acids. GAPDH is used as an indicator of RNA loading. (j) Western blot analysis from the above-mentioned livers was used to verify the degree of expression of peroxisomal and mitochondrial fatty acid metabolizing enzymes in wild-type and SRC knockout mice. Liver homogenates (20 *μ*g) from each group of mice were run on 4–20% SDS PAGE gel and immunoblotted using antibodies for peroxisomal (ACOX1, L-PBE, PTL, D-PBE, and SCPx) and mitochondrial (SCAD, MCAD, and VLCAD) fatty acid metabolizing enzymes. No difference in the induction was observed between SRC nulls and wild-type for the *β*-oxidation enzymes.

Mice lacking SRC-1 were generated to delineate its role in estrogen receptor, progesterone receptor, and PPAR*α* signaling [90, 92]. SRC-1 null mice are viable and fertile but show somewhat subdued response to sex hormonal stimuli after orchiectomy or ovariectomy [90, 96]. However, SRC-1^−/−^ mice when challenged with PPAR*α* ligands, such as Wy-14,643 or ciprofibrate, display the characteristic robust pleiotropic responses, including hepatomegaly, hepatic peroxisome proliferation and PPAR*α*-target gene activation [92]. These responses appear essentially similar to those exhibited by SRC-1^+/+^ littermates indicating that SRC-1 is not essential for PPAR*α* signaling in liver [92]. Likewise, as shown here, studies with SRC-2 and SRC-3 null mice also revealed that PPAR*α* target gene activation in liver is not dependent on these coactivators. Hepatic responses of SRC-1, SRC-2 and SRC-3 null mice following Wy-14,643 administration appear similar to those of wild-type mice treated with a PPAR*α* ligand (Figure 2). Histological evaluation of liver sections, processed to visualize peroxisomal catalase, show similar increases in the number of peroxisomes in hepatic parenchymal cells of wild-type and SRC null mice treated with a peroxisome proliferator (Figures 2(a)–2(h)). To further investigate the influence of SRC family on PPAR*α* function, we evaluated the changes in fatty acid-metabolizing enzymes in the liver of SRCs null and wild-type mice by Northern and Western blot analyses (Figures 2(i) and 2(j)). Northern blot analysis of total liver RNA shows similar basal levels of peroxisomal fatty acyl-CoA oxidase 1 (ACOX1), enoyl-CoA hydratase/L-3hydroxyacyl-CoA dehydrogenase bifunctional protein (L-PBE), peroxisomal 3-ketoacyl-CoA thiolase (PTL), and microsomal cytochrome P450 fatty acid *ω*-hydroxylase CYP4A1 in the livers of both wild-type and SRC-1, -2 and -3 null mice (Figure 2(i)). Massive increases in hepatic mRNA levels of these enzymes were noted in all SRC null mice treated with a PPAR*α* ligand (Figure 2(i)). The increases appear similar to that noted in the livers of Wy-14,643 treated wild-type mice (Figure 2(i)). Western blot analysis reveals increases in the content of fatty acid oxidation enzyme proteins in liver of intact and SRC null mice (Figure 2(j)). The expression levels of hepatic peroxisomal fatty acid *β*-oxidation enzymes ACOX1, L-PBE, D-PBE, PTL, and SCPx (sterol carrier protein x) were increased significantly after Wy-14,643 administration in both wild-type and SRC-1, -2, and -3 null mice (Figure 2(j)). Furthermore, no significant differences in the magnitude of increases are observed in hepatic mitochondrial enzymes short-chain acyl-CoA dehydrogenase (SCAD), medium-chain acyl-CoA dehydrogenase (MCAD), and long-chain acyl-CoA dehydrogenase (LCAD) among wild-type and SRCs null treated mice (Figure 2(j)). Together, these data indicate that no member of p160/SRC family of coactivators (SRC-1, SRC-2 and SRC-3) is required for PPAR*α*-mediated transcriptional activation in vivo [90]. 

Although the single gene-knockout mice have demonstrated that loss of individual members of p160/SRC family of coactivators is redundant for PPAR*α* function in liver, it remains to be ascertained if deletion of two or all three of these coactivators affects the PPAR*α* signaling. SRC-1 is required for the assembly of a complex that includes CBP/p300 for enhancing the coactivator function of PPAR*γ* coactivator-1 (PGC-1) [95]. It appears that the docking of PGC-1 to PPAR*γ* stimulates an apparent conformational change in PGC-1 that permits binding of SRC-1 and CBP/p300, resulting in a large increase in transcriptional activity, and this transcriptional enhancement function of PGC-1 fails to manifest in SRC-1 null cells [97]. SRC-1 null mice exhibit partial impairment of PPAR*γ* function with decreased PGC-1 regulated thermogenic activity in brown adipose tissue [95]. The lower energy expenditure in SRC-1 null mice predisposes to higher sensitivity to obesity upon high fat feeding [88, 95, 98]. Fatty acid oxidation in brown fat is also decreased due to partial impairment of PPAR*γ*/PPAR*α* function in the absence of SRC-1. 

SRC-2 has been implicated in a broader range of physiological processes including reproduction, mammary morphogenesis, uterine function and energy metabolism by affecting the regulation of adaptive thermogenesis [99]. SRC-2 null mice are viable, but the fertility of both sexes is impaired [94, 100]. These mice are resistant to diet-induced obesity and displayed enhanced adaptive thermogenesis [95]. In white adipose tissue, disruption of SRC-2 increases leptin expression as well as that of genes involved in lipolysis. Additionally, these mice manifest a decreased potential for fatty acid storage [95, 101]. Disruption of SRC-2 gene in the mouse reduces PPAR*γ* function in white adipose tissue resulting in a lesser degree of fat accumulation [76, 95]. This enhances the function and development of brown adipose tissue, leading to increased levels of uncoupling protein 1, PGC 1*α* and ACOX1, promoting energy expenditure [76, 87, 95]. Gene knockout studies have demonstrated that SRC-3 is required for normal growth, puberty and mammary gland development [93]. SRC-3 null mice also show reduced body weight and adipose tissue mass with a significant decrease in PPAR*γ* expression. At molecular level, SRC-3 interacts with the transcription factor CAAT/enchancer-binding protein(C/EBP) to control gene expression through PPAR*γ*. These results imply that SRC-3 exerts a key role in adipocyte differentiation in vitro and in vivo, and that this regulation of adipogenesis is upstream of PPAR*γ* [101, 102]. SRC-3 gene is often amplified or overexpressed in many types of cancers [93, 94]. 

The redundancy of p160/SRC family of coactivators in the energy balance or expenditure function of PPARs noted in SRC single-knockout mice suggests the existence of possible cooperative effects among the three members of the SRC family. To illuminate the physiological functions affected by double deletion of these coactivators, mice deficient in both SRC-1 and SRC-2 [100], or deficient in both SRC-1 and SRC-3 [50] have been generated. Most SRC-1/SRC-2 double null mice die at birth, generally before the weaning stage [100]. SRC-2^−/−^ mice are protected against obesity and display enhanced adaptive thermogenesis, whereas SRC-1^−/−^ mice are prone to obesity due to reduced energy expenditure. Together, these two members of SRC family control energy balance between white and brown adipose tissues through regulating PPAR*γ* activity [101]. 

Most SRC-1 and SRC-3 double null mice also die before birth and surviving combined-knockout mice are lean and resistant to high-fat diet induced obesity [50]. These mice exhibit a developmental arrest in interscapular brown adipose tissue and defective thermogenesis due to a deficiency in the regulation of selective PPAR*γ* target genes involved in adipogenesis and mitochondrial uncoupling. It is of interest that these double null mice consume more food because of lower leptin levels, but remain lean mostly due to a higher basal metabolic rate and enhanced physical activity [50]. Taken together, SRC-1 and SRC-3 play critical roles in energy balance by mediating both energy intake and energy expenditure [75].

To investigate the changes in gene expression profiles, microarray analysis has been used with the RNA from livers of SRC-1, SRC-2, and SRC-3 single null animals [98]. The overall pattern of altered hepatic gene expression in the SRC-1 null mice was one of upregulation as compared to wild-type mice. SRC-2 null mice appeared an overall downregulation compared to wild-type mice. In SRC-3 deficient mice, a minimal change of gene expression in liver was observed. All these data suggest that changes in gene expression for each SRC member show specific and nonoverlapping expression patterns and that the three members of SRC family play a key role in coregulating energy homeostasis and obesity [76, 98]. It is clear from experiments in mice that all three members of the SRC family contain both redundant and distinct functions and that each individual SRC contains the capacity to regulate different biological functions [53]. 

## 5. CBP/p300 with HAT Activity

CBP and p300, generally referred to as CBP/p300, are universal coactivators that link transcriptional activators to the basal transcription apparatus and provide a scaffold to integrate multiple cofactors. CBP was first identified as a protein that binds CREB (cAMP response element-binding protein), whereas p300 was cloned as an adenovirus oncoprotein E1A-associated protein [56]. Subsequent studies established that CBP and p300 are significantly related and that human CBP resembles the human p300 more closely [103, 104]. These proteins are well conserved amongst mammals and homologs of CBP/p300 have been found in *Drosophila*, *Caenorhabditis elegans*, and *Arabidopsis thaliana *[105]. No CBP/p300 homologs are found in prokaryotes or yeast [104]. CBP/p300 proteins possess HAT activity and the HAT domain has the ability to recruit other proteins such as p160/SRC family members with HAT activity to further enhance the acetylation potential of the coactivator complex to remodel chromatin structure for efficient gene transcription [76, 106–108]. CBP/p300 proteins share several conserved regions, which constitute most of their known functional domains such as bromodomain [109], cysteine/histidine-rich domains, a KIX domain to which transcription factor CREB binds, glutamine- and proline-rich domain, receptor-interacting domain, and SRC-1 interaction domain [108]. The C-terminal glutamine-rich domain of CBP/p300 forms contacts with other coactivators, most notably those involved in nuclear hormone-receptor signaling pathways. The complexity and breadth of CBP/p300 interactions attest to the unique involvement of CBP/p300 in the transcriptional control as universal and versatile cointegrators. 

CBP and p300 directly interact with the ligand-binding domain of several nuclear receptors including PPARs [110]. C-terminal of PPAR*α* that corresponds to AF-2 domain (residues 448–468) is required for the interaction with N-terminal region of p300 spanning aa 39–117, and also the N-terminal fragment of CBP encompassing aa 1–115 in a ligand dependent manner. Fragments of both p300 and CBP that interact with PPAR*α* contain one LXXLL motif [110]. Interaction of CBP and p300 with the ligand-binding domain of PPAR*α* or PPAR*β* was demonstrated in human intestine-like Caco2 cell line [111]. Induction of conformational change and transactivation potential of PPAR*β* was considerably lower than that of PPAR*α* in response to arachidonic acid, as well as other polyunsaturated fatty acids [112]. Arachidonic acid enhanced binding of p300 to PPAR*α* but not to PPAR*β*. Additionally, arachidonic acid induced in vitro binding of both PPAR*α*-RXR*α* and PPAR*β*-RXR*α* heterodimers to several PPREs [113, 114]. CBP, which is highly expressed in brown fat, also coactivates PPAR*α*-dependent regulation of the UCP-1 gene promoter in the HepG2 cells in the presence of PPAR*α* ligand, Wy-14,643 [115]. Presence of CBP in PRIC complex that interacts with full-length PPAR*α* in the presence of ciprofibrate and leukotriene B4 also substantiate the coactivator status of CBP [63]. 

p300 interacts with the N- and C-terminal regions of PPAR*γ* in a ligand-independent and -dependent manner, respectively [116]. Leu-311, Asn-312, and Thr-316 in helix 4 of PPAR*γ* ligand-binding domain are involved in PPAR*γ* binding with CBP [117]. Deletion of A/B-domain of PPAR*γ* compromises recruitment or stabilization of CBP- and p300-containing cofactor complexes on a subset of target genes involved in lipid storage [118]. Both PPAR*γ* and CBP are expressed in preadipocytes and differentiated adipocytes suggesting that CBP serves as a physiologically relevant coactivator for PPAR*γ* signal transduction [119]. p300 also transactivates PPAR*γ* in the presence of natural ligand 15-deoxy-Δ12, 14-prostaglandin J2, but troglitazone, a synthetic PPAR*γ* ligand, failed to induce PPAR*γ* interactions with p300. CBP/p300 also increases the transcriptional activity of PPAR*γ* through PGC-1 which stimulates an apparent conformational change in PGC-1 that permits binding of SRC-1 and CBP/p300 resulting transactivation of target genes [91]. Recruitment of CBP/p300 and PGC-1*α* was shown recently on PPAR*γ*/RXR*α* heterodimer bound to the promoter of UCP-1 gene after the activation of *β*-adrenergic receptor in Jhdm2a knockout mice [120]. Jhdm2a is a H3K9-specific demethylase that directly regulates PPAR*α* and Ucp1 expression [120]. PPAR*γ* recruits CBP to the aP2 gene promoter as evidence by chromatin immunoprecipitation and in vitro immunoprecipitation assay in the MEFs induced for adipogenic differentiation [121]. Recently, it has been shown that beraprost, a synthetic analogue of prostacyclin serves as a ligand for PPAR*β* that enhances transcriptional activation of p21/p27 by increasing CBP nuclear translocation, which contributes to the vasoprotective action in rat aortic smooth muscle cells [122]. 

## 6. PBP (PPARBP)/TRAP220/DRIP205/MED1

PPAR-binding protein (PBP/PPARBP) was first cloned through yeast two-hybrid system using Gal4-PPAR*γ* as bait to screen a mouse liver cDNA library and identified as a nuclear receptor coactivator with 2 LXXLL motifs [58]. Subsequently, PBP was shown as a critical component of TRAP/DRIP/ARC/Mediator complex [57, 73, 74, 123, 124] and it is variously referred to as PBP (PPARBP)/TRAP220/DRIP205/Med1 subunit of Mediator complex [41]. The Mediator complex was first discovered in yeast and was shown to be essential for pol II dependent transcription [41]. The mammalian Mediator complex consists of ~31 subunits and PBP/TRAP220/Med1 is the prominent member of this complex [73, 75]. Med1 binds to several nuclear receptors such as PPAR*α*, RAR*α*, TR*β*1, RXR, VDR, FXR, ER*α* and GR via two conserved LXXLL motifs in a ligand-dependent manner [58, 74, 121, 123–125], Med1 also interacts with a variety of other transcriptional factors, including tumor suppressor p53, five GATA family members, p300, PGC-1 and C/EBP*β* [46, 79, 126–129]. These interactions imply a major role for Med1 in nuclear receptor mediated cellular proliferation, differentiation and homeostatic regulation [76, 130]. 

Med1 serves as an anchor for the Mediator complex and facilitates the linkage between HAT containing CBP/p300 and p160/SRC protein complex and pol II basal transcription machinery in regulating transcription [41, 46]. Phosphorylation of Med1 by mitogen-activated protein kinase- extracellular signal-regulated kinase (MAPK-ERK) promotes its association with Mediator [131, 132]. Med1 is widely expressed in many tissues of adult mice, including brain, heart, lung, liver, kidney, adipose tissues, and the most prominent being the testis [76, 133]. Recently, it has been reported that Med1 is a target for miR-205. miR-205 interacts with a specific target in the 3′-UTR sequence of Med1 and silences its expression in human trophoblasts exposed to hypoxia [134]. 

Med1 ablation leads to embryonic lethality at midgestation, day 11.5 postcoitum (E11.5), which is attributed, in part, to defects in the development of placental vasculature, similar to those encountered in PPAR*γ* [78, 135–137]. Embryonic development of the heart, eye, vasculature and the hematopoietic system is altered in Med1 null mice. This phenotype is similar to that in mice deficient in members of GATA, a family of transcription factors that modulates differentiation of adipocytes, megakaryocytes and erythrocytes [79]. 

As indicated above, Med1 was first identified as a PPAR*γ* coactivator and it plays an important role in the PPAR*γ* signaling pathway [58, 78, 136]. Med1 and PPAR*γ* interaction requires two LXXLL nuclear receptor recognition motifs present in Med1 [138]. Med1 modestly increases the transcriptional activity of PPAR*γ*, and a truncated form of Med1 (aa 487–735) acts as a dominant negative repressor [74, 123]. It has been shown that the deletion of 12 amino acids from the extreme carboxyl terminus of PPAR*γ* results in the abolition of Med1-PPAR*γ* interaction [58]. However, deletion of the PPAR*γ* A/B-domain does not affect Med1 recruitment [118]. To study the role of Med1 in PPAR*γ*-mediated adipogenesis in vitro, Med1^+/+^ and Med1^−/−^ MEFs were isolated from E10.0 littermate embryos and infected with a retroviral vector driving PPAR*γ* [139]. Disruption of TRAP220/Med1 in MEFs is refractory to PPAR*γ*-stimulated adipogenesis but not MyoD-stimulated myogenesis [139]. Surprisingly however, a conserved N-terminal region of Med1 that lacks the LXXLL motifs but gets incorporated into Mediator fully supports PPAR*γ*-stimulated adipogenesis [138]. A direct interaction between PPAR*γ* and the mediator complex through Med1 is not essential for PPAR*γ*-stimulated adipogenesis and for PPAR*γ* target gene expression in cultured fibroblasts [138]. Furthermore, PPAR*γ* target gene expression and recruitment of Mediator to a PPAR*γ* response element on the aP2 promoter in undifferentiated MEFs do not require Med1 [138]. These findings imply that the presence of alternative mechanisms for Mediator recruitment, possibly through intermediate cofactors or other cofactors that are functionally redundant with Med1 [138]. 

**Figure 3:**
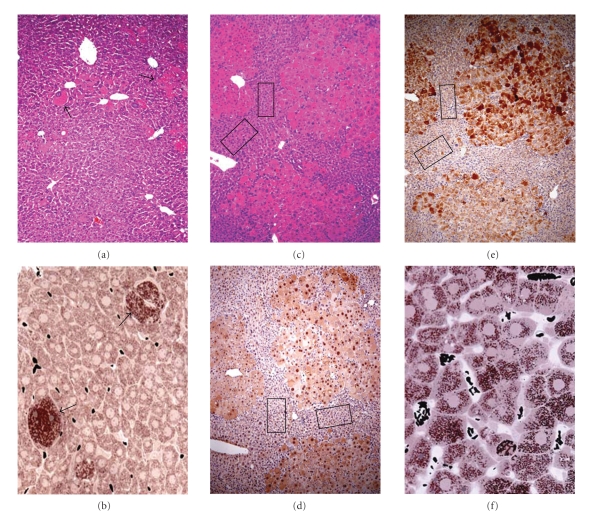
Effects of Med1 and PRIP deletion on PPAR*α* ligand-induced pleiotropic responses in liver. Med1^ΔLiv^ mice treated with Wy-14,643 (0.125% wt/wt) for 2 weeks (a, b) show an occasional hepatocyte that escaped Alb-Cre mediated deletion of Med1 floxed alleles. These Med1 positive hepatocytes respond to the peroxisome proliferative effects of PPAR*α*-ligand (arrows indicate to Med1^+/+^ cells with numerous peroxisomes) but not the majority of Med1^−/−^ hepatocytes. Chronic treatment of Med1^ΔLiv^ mice with 0.02% Wy-14,643 for 5 months (c–e) results in clonal expansion of residual Med1^fl/fl^ cells as demonstrated by H&E staining (c). In contrast, the adjacent hepatocyte lacking Med1 are generally smaller than normal hepatocytes (see boxed area in c). Immunohistochemical localization of Med1 reveals that expanding colonies of large hepatocytes are Med1 positive (nuclear Med1 in panel d). These cells also show abundant cytoplasmic expression of L-PBE, the second enzyme of the peroxisomal fatty acid *β*-oxidation system (panel e), whereas the smaller Med1 null hepatocytes (*boxed areas*) fail to show L-PBE induction (panel e). Disruption of coactivator PRIP in hepatocytes does not interfere with PPAR*α* ligand-induced peroxisome proliferation as evidenced by the abundant catalase positive peroxisomes (brown dots) in all hepatocytes (panel f). Compare this panel (f) with panel (b) in which only an occasional Med1^+/+^ cell responds to PPAR*α* ligand.

To further study the role of Med1 in specific tissues in vivo, mice carrying floxed Med1 alleles were generated for conditional null mutation [140, 141]. Conditional deletion of Med1 gene in liver results in the abrogation of PPAR*α* ligand-induced pleiotropic effects, indicating that Med1 is essential for PPAR*α* signaling and fatty acid oxidation [140]. Med1 deficiency in liver parenchymal cells results in the near abrogation of PPAR*α* ligand-induced peroxisome proliferation, liver cell proliferation, and induction of PPAR*α*-regulated genes. In contrast, scattered residual Med1^+/+^ hepatocytes that escape Cre-mediated excision of floxed alleles in Med1 liver nulls, show DNA synthesis and were markedly hypertrophic with peroxisome proliferation in response to PPAR*α* ligands (Figures 3(a) and 3(b)). Med1^−/−^ hepatocytes are refractory for PPAR*α* ligand-induced peroxisome proliferation [140]. Moreover, Med1^ΔLiv^ mice, chronically exposed to PPAR*α* ligand Wy-14,643, show a striking proliferative response and clonal expansion of residual Med1^+/+^ hepatocytes (Figures 3(c) and 3(d)) but no proliferative expansion of Med1^−/−^ hepatocytes occurs and these Med1 null hepatocyte appeared hypoplastic (boxed areas in Figures 3(c)–3(e)) as compared to hyperplastic large Med1^+/+^ hepatocytes. 

Surprisingly, the Med1 liver conditional null mice develop liver tumors on long-term exposure to PPAR*α* ligand, but all tumors developing in Med1^ΔLiv^ mice reveal Med1 expression and no tumors developed from Med1^−/−^ hepatocytes [142]. These data suggest that Med1 plays a key role in PPAR*α* ligand-induced liver tumor development and that cells deficient in Med1 do not give rise to tumors [142]. Furthermore, initiation by a genotoxic carcinogen diethylnitrosamine followed by phenobarbital promotion in Med1^ΔLiv^ mice results in a failure of Med1 null hepatocytes to undergo proliferation. As in the case of Wy-14,643 treatment, all hepatocellular carcinomas developing in Med1^ΔLiv^ mice are Med1 positive [143]. Liver tumors that develop in Med1^ΔLiv^ mouse livers are transplantable in athymic nude mice and these maintain Med1^fl/fl^ genotype. These observations imply that Med1 is essential for the development of hepatocellular carcinoma in the mouse [143]. The failure of Med1 null hepatocytes to develop liver tumors following PPAR*α* ligand administration or after prolonged promotion with phenobarbital, which is an activator for nuclear receptor constitutive androstane receptor (CAR), implies that coactivator Med1 is a critical component of PPAR*α* and CAR signaling and thus participates in the neoplastic process [142–144]. Med1 deficient livers fail to develop hepatic steatosis induced by glucorcorticoid receptor (GR) agonist [145] and also fail to develop hepatic steatosis when induced by PPAR*γ* overexpression (unpublished data). In addition, using a conditional null mutation, it has been shown that Med1 is required for mammary gland development [146], and is also essential for the growth of Notch4-immortalized mammary cells by activating SOX10 expression [147]. Earlier studies have demonstrated the Med1 is either overexpressed or amplified in several breast carcinomas implying that Med1 plays a role in ER signaling and cancer [125, 148]. More recently, Med1 has been shown to play an important coregulatory role in prostate cancer cell proliferation and survival [149]. However, decrease of Med1 expression in human melanoma cells increases their tumorigenic phenotype and the reason for this discordancy is unclear [150]. 

 In summary, using conditional knockout mice, it has been established that Med1 subunit is essential for the signaling of nuclear receptors PPAR*γ*, PPAR*α*, CAR and GR [139–145]. Evidence indicates that Med1-deficiency does not lead to the disintegration of the Mediator complex as originally speculated but it is possible that Mediator complex devoid of Med1 subunit may be impaired in its ability to recruit pol II to transduce the transcriptional signal [151]. 

## 7. PGC-1 Family in Coactivation of PPAR

PGC-1 family of coactivators, with three members, plays a critical role in the maintenance of mitochondrial function, thermogenesis and energy homeostasis [59]. The first member of the PGC-1 family was identified as a PPAR*γ*-interacting protein from brown fat cDNA library using yeast two-hybrid screen and is now termed PGC-1*α* [59]. Thereafter, two related coactivators, PGC-1*β* (also termed PERC) and PGC-1-related coactivator (PRC) were discovered through searches of new data base entries [60, 152]. PGC-1*α* and PGC-1*β* share similar tissue distribution with highest levels of expression in brown fat, heart and slow-twitch skeletal muscle [59, 152], and their mRNA levels are induced significantly in the adult liver following fasting [152, 153]. Expression of PGC-1*α* mRNA is also elevated in brown fat after cold exposure, whereas PGC-1*β* does not respond [59, 152]. Less is known about the expression patterns and biological roles of PRC [60]. 

In addition to PPAR*γ*, PGC-1*α* also coactivates a variety of other nuclear receptors, including PPAR*α* [154], PPAR*β* [21], TR*β* [59], ER*α* [59], GR [155], FXR [156], LXR [157], HNF4 [152] and RAR, but not RXR*α* [59]. Cotransfection experiments in cells show that PGC-1*α* increases the PPAR*α*-mediated transcriptional activity and that AF2-LXXLL interaction is necessary for the coactivation of PPAR*α* by PGC-1*α* [154]. Furthermore, overexpression of PPAR*α* and PGC-1*α* in 3T3L1 cells cooperatively induces the expression of mitochondrial fatty acid *β*-oxidation enzyme system genes and increases cellular palmitate oxidation rates [154]. PPAR*α*-driven mitochondrial biogenic response reveals that expression of PGC-1*α* is activated in wild-type mice but not in PPAR*α*-deficient mice [158]. PGC-1*α* promotes expression of mammalian tribbles homolog TRB-3 through PPAR*α* and knockdown of hepatic TRB-3 expression improves glucose tolerance, whereas hepatic overexpression of TRB-3 reversed the insulin-sensitive phenotype of PGC-1-deficient mice [159]. 

PGC-1*α* utilizes a domain rich in proline residues to bind to a region that overlaps the DNA binding and hinge region of PPAR*γ* [59]. The strong interaction of PGC-1*α* and PPAR*γ* is mediated through both hydrophobic and specific polar interactions. Mutations within the context of the full-length PGC-1*α* indicate that the first PGC-1*α* motif is necessary and sufficient for PGC-1*α* to coactivate PPAR*γ* in the presence or absence of rosiglitazone [160]. Thiazolidinediones and rexinoids induce PGC-1*α* gene expression in brown and white adipocytes by a PPAR*γ*-dependent pathway. This is attributed to the presence of a PPAR*γ*-responsive element in the distal region of the PGC-1*α* gene promoter that binds PPAR*γ*/RXR heterodimers [161]. The interaction between PGC-1*α* and PPAR*β* depends on the LXXLL motif in PGC-1*α* (aa 142–146) and this interaction is enhanced in the presence of PPAR*β* agonist GW501516 [21]. Tetradecylthioacetic acid, a pan-PPAR ligand, induces hepatic fatty acid oxidation in PPAR*α* ^−/−^ mice possibly through PGC-1*α* mediated PPAR*β* coactivation [162]. Pharmacological activation of PPAR*β* induces fatty acid oxidation and this depends upon PGC-1*α* as the induction is completely abolished in the absence of both PGC-1*α* and PGC-1*β* [163]. To study the role of PGC-1*α* in vivo, PGC-1*α* knockout mice have been generated [164, 165], which show normal embryonic development, suggesting that PGC-1*α* driven coactivation is not critical for PPAR*γ* and PPAR*β* functions in the maintenance of placental adequacy [164, 165]. Interestingly, PGC-1*α* ^−/−^ mice survive with modestly blunted postnatal cardiac growth, suggesting that PGC-1*α* is essential for the maintenance of maximal, efficient cardiac mitochondrial fatty acid oxidation, ATP synthesis, and myocardial lipid homeostasis [165, 166]. 

Hepatic PGC-1*β* overexpression results in the attenuation of changes induced by Wy-14,643, a PPAR*α* ligand [167]. PGC-1*β* poorly activates the expression of gluconeogenic genes in hepatocytes or liver in vivo. The reduced ability of PGC-1*β* to induce gluconeogenic genes is due, in part, to its inability to physically associate with and coactivate HNF4*α* and FOXO1 [153]. PGC-1*β* null mice are viable and fertile and show no overt phenotype. However, PGC-1*β* deficient mice display an altered expression in a large number of nuclear-encoded genes governing mitochondrial functions in multiple tissues including heart, skeletal muscle, brain, brown adipose tissue, and liver [168]. PGC-1*α* null mice appeared hyperactive in comparison to somewhat sluggish PGC-1*β* null mice. When acutely exposed to cold, these mice develop abnormal hypothermia and morbidity [168]. Furthermore, high-fat feeding induced hepatic steatosis and increases serum triglyceride and cholesterol levels in the mutant mice [168]. 

## 8. PRIP/NCoA6

Nuclear receptor coactivator PRIP (PPAR-interacting protein) [168], also referred to as activating signal cointegrator-2(ASC-2) [83]/nuclear receptor activating protein 250 (RAP250) [61]/nuclear receptor coregulator (NRC) [84]/thyroid hormone receptor (TR)-binding protein (TRBP) [85], was cloned by different groups using yeast two-hybrid screens with a nuclear receptor as bait. PRIP (NCoA6) was identified as a ligand-dependent nuclear receptor-interacting protein. PRIP forms large steady-state complex of approximately 2MDa (ASC-2 complex [ASCOM] with retinoblastoma-binding protein RBQ-3, *α*/*β*-tubulins and subset of Trithorax-related proteins [169]. PRIP, like MED1 gene is also amplified and overexpressed in breast, colon and lung cancers [170, 171]. PRIP and PRIP-interacting protein with methyltransferase domain (PIMT/NCoA6IP) appears to serve as a linker between CBP/p300-anchored and Mediator complexes. PRIP contains two LXXLL motifs, one at the N-terminal region (aa 892 to 896) plays a pivotal role for ligand-dependent interactions with a wide spectrum of nuclear receptors including PPARs, TRs, RXRs, ERs, GR, VDR and RARs [64, 81, 83] and the second LXXLL located at the C-terminal does not bind with PPARs, RAR, RXRs, and GR but does bind with LXR*α* and LXR*β* [85, 172–174]. These two LXXLLs interact with LXRs and other nuclear receptors and regulate insulin secretion and maintain *β*-cell function [175]. 

PRIP acts as a strong coactivator for PPAR*γ*, and a truncated form of PRIP (aa 786–1132) acts as a dominant-negative repressor [62]. It is worth noting that PRIP is also detected in the transcriptionally active PPAR*α* interacting cofactor (PRIC) complex isolated from rat liver [63]. PRIP protein expression has been detected in many tissues, especially in the reproductive organs such as testis, prostate and ovary [76, 176]. In the testis and ovary, PRIP immunostaining shows intense staining of the nuclei of Sertoli and follicular granulosa cells respectively [177]. Mice with disrupted PRIP/RAP250/NRC/AIB3 gene die at embryonic stage E11.5 and E12.5 days. PRIP mutant embryo mortality has been attributed to placental dysfunction including the failure of labyrinthine development, the dilation of maternal blood sinuses, the massive erythrophagocytosis by trophoblastic cells, alteration in trophoblast population and the formation of fewer blood vessels in extra-embryonic membrane covering the embryo [76, 85, 170, 173, 178, 179]. In addition, developmental abnormalities in heart, liver, and the nervous system have been noted [85, 173, 178]. Interestingly, MEFs derived from PRIP/NRC null embryos display growth retardation and apoptosis [176]. Further studies with heterozygous PRIP/NRC^+/-^ show a spontaneous wound healing deficiency, suggesting that PRIP/NRC is important in maintaining integrity during wound healing [176, 180]. Haploid inactivation of PRIP/AIB3 in AIB3^+/-^/PyMT bitransgenic mice cause inhibition of cell proliferation mediated by PPAR*γ*/RXR [181].

PRIP null MEFs are also resistant to PPAR*γ* stimulated adipogenesis [121]. This defect occurs because of apparent disruption of the linkage between the CBP/p300 anchored subunit complex and the MED1-dependent mediator complex [121]. In order to investigate the physiological role of PRIP in vivo, conditional knockout mice have been generated [182]. Conditional PRIP null mutation in the mouse mammary gland results in defective mammopoiesis, similar to that encountered in Med1 deficient mammary glands [146, 168]. To further understand the function of PRIP in mammary gland tumorigenesis, a mammary tumor cell line with the PRIP^loxP/loxP^ genotype was established and disruption of the PRIP gene in these cells has been shown to abrogate their tumorigenic potential. PRIP deficiency substantially reduced the expression of FOS gene [171]. Liver-specific disruption of the PRIP gene fails to affect the induction of PPAR*α*-regulated pleiotropic responses, including hepatomegaly, hepatic peroxisome proliferation (Figure 3(f)), and induction of genes involved in the fatty acid oxidation systems [182]. These results are dissimilar to those encountered with liver specific MED1 gene disruption [140], indicating that PRIP is not essential for PPAR*α* target gene activation in liver [182].

## 9. PRIC285

PRIC complex isolated from the rat liver nuclear extract using full-length GST-PPAR*α* fusion protein in the presence of a PPAR*α* ligand comprises of ~25 subunits [63]. A protein complex similar to this was also obtained using PPAR*α* ligand (ciprofibrate)-affinity matrix [64]. MALDI-TOF analysis of the components of PRIC complex showed identities of many already known genes identified in yeast two-hybrid screens known to be involved in transcriptional regulation and some novel proteins [63]. This complex includes CBP/p300, p160/SRC-1, MED1, PRIP, PIMT and novel coactivators designated as PRIC285, PRIC295, and PRIC320 based on estimated molecular size (see [63, 64], unpublished data). PRIC285 is a component in the PRIC complex isolated using ciprofibrate or LTB4 as ligand in GST PPAR*α* pull down system [63]. Subsequently, a longer isoform has been cloned using human PPAR*γ* as bait in yeast two-hybrid screen and has been referred to as PPAR*γ*-DBD-interacting protein 1 (PDIP1)-*α* [183]. PRIC285 is expressed in multiple human tissues such as skeletal muscle, colon, spleen, liver, kidney, heart, lung, pancreas, small intestine, thymus, prostate, ovary, peripheral blood, and placenta [63]. PRIC285 has been detected in several human cancer lines such as HeLa, colorectal adenocarcinoma SW480, melanoma, HepG2, medulloblastoma HTB185, and DU145 (prostate) [63, 183]. 

The human PRIC285 gene, which spans ~16.1 kb, is located on chromosome 20 at position 20q13.33 and it encodes a protein of 2080 amino acid with an estimated molecular mass of 285 kDa [63]. PRIC285 contains five LXXLL signature motifs at aa 506–510; 549–553; 604–608; 1443–1447; 1660–1664 [183]. It appears that none of these LXXLL motifs of PRIC285 is needed for interaction with PPARs as demonstrated by mutating LXXLL motifs [183]. PRIC285 binds to the DBD-hinge (DBD-H) of the PPARs through its C-terminal region mapped at aa 1675–1823 [183]. Comparison of the amino acid sequences flanking core LXXLL motifs in PRIC285 with those identified in other coactivators revealed that this configuration did not fit well with the proposed alignment rules [184]. Other than LXXLL signature motifs human PRIC285 also displays amino acid sequence homologous to RnaseB (RNB) and UvrD/REP motifs, a superfamily I DNA helicase [63, 183]. It is found to be transcriptional coactivator for the PPAR*α*, PPAR*δ*/*β* and PPAR*γ* in transfected cells. Cotransfection of PPAR*α* and PRIC285 into HEK293 cells stimulates transcription of PPRE-TK-Luc gene in the presence of ciprofibrate, a PPAR*α* specific ligand. This interaction between PRIC285 and PPAR*α* was also shown by the colocalization in nucleus of 293 cells [63]. Transactivation of PPAR*α* by PRIC285 also occurs after treatment with a different PPAR*α* ligand, fenofibrate [183]. Human PRIC285 has been shown to enhance PPAR*γ*-mediated transactivation of DR1 reporter gene using the synthetic PPAR*γ* ligand troglitazone. PRIC285 also coactivates PPAR*β* in the transactivation assay where CV-1 cells were treated with PPAR*β* ligand cyclic prostaglandin [183]. 

To assess the biological significance of PRIC285, we have generated whole-body gene knockout mice using two-*loxP* and two-*frt* system and characterized them for PPAR*α*-mediated transcriptional activation in vivo [185]. Mice homozygous for PRIC285 mutation (PRIC285^−/−^) are apparently healthy and fertile and show no consistent phenotypic differences when compared to their wild-type floxed littermates (PRIC285^fl/fl^). When challenged with PPAR*α* ligands, such as Wy-14,643 or ciprofibrate, no differences were observed in the magnitude of pleiotropic responses, which include hepatomegaly, peroxisome proliferation in hepatocytes, and increased levels of PPAR*α* target genes such as peroxisomal and mitochondrial *β*-oxidation enzymes [185]. The role of PRIC285 in PPAR*γ* mediated adipogenesis in the liver has been examined using PRIC285 null mice. Adenovirally driven PPAR*γ* gene when injected through tail vein induced hepatic steatosis in both PRIC285 null and wild type floxed littermates to delineate the role of the coactivator PRIC285 in hepatic steatosis. No discernible differences in the PPAR*γ*-mediated hepatic adipogenic steatosis in the normal and mutant PRIC285 mouse liver has been noted [L. Bai, unpublished]. These results may point to a functional redundancy of PRIC285 in the general transcriptional machinery as far as PPAR*α* and PPAR*γ* are concerned. The discordance between in vitro and in vivo results of PRIC285 function reflects the complexity and redundancy in that loss of a single component of a multisubunit protein complex could be compensated in vivo by other members of this mega complex. Nonetheless, it would be a challenge for the immediate future to assess the role of PRIC285 in the signaling of other nuclear receptors in vivo using the whole-body or conditional deletion. 

## 10. PRIC320/CReMM/CHD9: ATP Dependent Chromatin Remodeling Activity

PRIC320 was identified in high molecular weight protein complex isolated using ciprofibrate coupled AH-sepharose affinity pulldown from the rat liver nuclear extract [64]. PRIC320 is also known as chromodomain helicase DNA binding protein 9 (CHD9)/Chromatin Related Mesenchymal Modulator (CReMM). It is a member of the CHD (chromodomain-helicase-DNA-binding) family of proteins that interacts with nucleosomes and plays a role in chromatin remodeling to modulate transcription [64, 186–188]. Members of the CHD family of enzymes belong to the SWI/SNF2 (SWItch/Sucrose Nonfermentable) superfamily of ATP-dependent chromatin remodelers. PRIC320 displays two tandem N-terminal chromodomains (aa 692–752; 774–826) that function as interaction surfaces for a variety of chromatin components. It also contains SNF2-like ATPase/DEAD-like helicase domain (aa 879–1028) located in the central region of the protein structure [189, 190]. A C-terminal cluster of domains such as paired BRK (Brahma and Kismet; aa 2483–2532; 2557–2601) domains, a SANT-like domain, and a DNA-binding domain are also present in PRIC320 [64, 191, 192]. The SNF2-like ATPase/DEAD-like helicase domain contains a conserved set of amino acid motifs that has been found in proteins involved in many of cellular processes including chromatin assembly, transcription regulation, DNA repair, DNA replication, development and differentiation [69, 193]. PRIC320 contains five LXXLL signature motifs that mediate interaction with nuclear receptors [64]. 

Two isoforms of PRIC320 designated as PRIC320-1 and PRIC320-2 that encode aa 2882 and 1995, respectively, have been identified in the human [64]. PRIC320-1 with an estimated molecular weight of 320 kDa contains all five LXXLL motifs located at aa 868–872; 1036–1040; 2031–2035; 2706–2710; and 2778–2782, whereas PRIC320-2 with an estimated molecular weight of 240 kDa contains distal four LXXLL motifs. The gene encoding human PRIC320 is mapped to long arm of chromosome at 16q12.2 (Ensembl; www.ensembl.org). PRIC320 transcript is present in various human tissues though at very low levels. PRIC320 mRNA has been detected in cancer cell lines such as HL-60, HeLa, Burkitt's lymphoma Raji and colorectal and lung carcinoma [64]. Cancer cell lines such as HL-60, HeLa cells, are shown to express two isoforms of mRNA 11.5 kb and 10.5 kb corresponding to PRIC320-1 and PRIC320-2. In mice, expression of PRIC320 is higher in brain, followed by heart, kidney, and skeletal muscle [64]. 

PRIC320 interacts with PPAR*α* and functions as a coactivator in vitro [64]. Full length PPAR*α* fused to GST interacts with both PRIC320 isoforms in a ligand dependent manner whereas interaction with PPAR*γ* appeared minimal [64]. This selectivity for PPARs indicates a differential role of PRIC320 in the regulation of downstream target genes. The recognition in PRIC320/CHD9 of chromatin remodeling function and nuclear receptor coactivator function is suggestive of the multiple roles played by these nuclear receptor cofactors. 

## 11. SWI/SNF: ATP-Dependent Chromatin Remodeling Complex

The SWI/SNF (mating type switch/Sucrose Nonfermenting) families of chromatin remodeling complexes mobilize nucleosomes and function as master regulators of transcription factor function. Although the precise mechanisms by which SWI/SNF modifies chromatin structure remains unclear, this process involves a conformational change of nucleosome and chromatin-remodeling in an ATP-dependent manner [65, 66]. SWI/SNF complex contains one or two possible ATPases, BRM (Brahma) or BRG1 (Brahma-Related gene 1) [194]. Chromatin remodelling represents an important step in adipocyte differentiation. C/EBP*α*, which is known to interact with the pol II-associated general transcription factors TBP/TFIIB, also interacts with BRM of the human SWI/SNF complex [195]. PPAR*γ* depends on a specific BRG1-containing SWI/SNF complex to activate adipogenesis under in vitro conditions [196]. SWI/SNF complex and TFIIH are recruited on the promoter of PPAR*γ* to transactivate PPAR*γ* [197]. The docking of SWI/SNF complex on PPAR*γ* promoter occurs through the subunit BAF60c (BRG1/Brm-associated factor subunit c) [69]. Recently, an interaction between SWI/SNF complexes and PPAR*α* was demonstrated through BAF60a. SWI/SNF also plays a role in the regulation of the hepatic lipid metabolism through the fatty acid oxidation [67]. 

## 12. BAF(s) Family

The BAF (BRG1/Brm-associated factor) family represents the accessory subunits of SWI/SNF complexes that act as the connection between transcription factors and SWI/SNF complexes. Several BAFs been identified which include BAF250, BAF170, BAF155, BAF60, BAF57 and BAF53a [198–200]. Recent studies have implicated the BAF60 family members, including BAF60a, BAF60b and BAF60c, in mediating the interaction between the SWI/SNF complexes and target transcription factors. BAF60a or SMARCD1 (SWI/SNF related, matrix associated, actin-dependent regulator of chromatin subfamily d, member 1), a protein of 60 kDa, is known to be the connection between SWI/SNF and GR [68]. More recently, BAF60a was identified as a molecular link between SWI/SNF complexes and hepatic lipid metabolism. Adenoviral expression of BAF60a has been shown to stimulate fatty acid *β*-oxidation in primary hepatocytes culture to ameliorate hepatic steatosis in vivo. PGC-1*α* mediates the recruitment of BAF60a to the PPRE and enhances the transcription of PPAR*α* regulated fatty acid oxidation system genes [67]. BAF60a is considered as a regulator of hepatic lipid metabolism. BAF60c or SMARCD3 (SWI/SNF related, matrix associated, actin dependent regulator of chromatin subfamily d, member 3), which is also a 60 kDa protein, binds to several nuclear receptors, including PPAR*γ*, ER*α*, and ROR*α* [69]. Recently, a new regulator of PPAR*γ*, the subunit BAF60c2 has been identified in a yeast two-hybrid screen of a human adipose tissue cDNA library. BAF60c2 represents a new isoform of BAF60c, which allows the recruitment of SWI/SNF to the nuclear receptor. Two isoforms BAF60c1 and BAF60c2 are localized primarily in the nucleus and are expressed in a wide variety of tissues. BAF60c proteins interact in a ligand-independent manner with PPAR*γ* and enhance its transcriptional activity [69].

## 13. PIMT

PIMT (NCoA6IP, TGS1) was first isolated as a PRIP (NCoA6)-interacting protein [70]. PIMT is an RNA methyltransferase and was cloned from a human liver cDNA library using PRIP as bait in yeast two-hybrid assay. Human PIMT protein contains 852 amino acids. It has a methyltransferase motif at the C- terminus and an RNA-binding domain at the N-terminus [70]. PIMT is an evolutionarily conserved protein found in C. elegans, Arabidopsis thaliana, and yeast [70]. PIMT serves as a linker between multiprotein complexes anchored by CBP/p300 and PBP/MED1. PIMT enhances Med1mediated transcriptional activity of the PPAR*γ* which was increased by PRIP [70, 79, 201]. Consistent with its RNA methyltransferase function, PIMT homologue in yeast known as trimethylguanosine synthase1 (TGS1), plays a role in the formation of the 2, 2, 7-trimethylguanosine (m3G) 5′-cap structure of snRNAs and small snoRNAs [202]. In Drosophila it is designated as DTL (Drosophila Tat-like) where it is important in development [203]. PIMT is localized predominantly to the nucleus. It is expressed in most adult tissues and in all embryonic stages in the mouse [76]. Inhibition of PIMT by siRNA in HeLa cells results in G2/M phase arrest [204]. 

In order to investigate the biological functions of this gene in mammalian development and growth, PIMT gene knockout mice have been generated [205]. Heterozygous (PIMT^+/-^) mice grow normally and are indistinguishable from their wild-type (PIMT^+/+^) littermates. Disruption of both PIMT alleles results in early embryonic lethality due to apoptosis and decreased proliferative potential of the blastocyst cells [205]. PIMT deficient embryos die shortly after implantation and then resorbed. PIMT^fl/fl^ MEFs treated with adenovirus expressing Cre showed defective wound healing and G2 phase arrest of cell cycle. These results suggest that PIMT is important for early embryonic development of mice [205]. 

## 14. CARM1

Protein methylation is involved in regulating protein-protein interactions that affect key cellular events, including regulation of transcription [206]. Proteins can be methylated irreversibly on the side-chain nitrogens of the amino acids arginine, lysine, and histidine in a reaction with S-adenosylmethionine (AdoMet) [207]. Coactivator-associated arginine methyltransferase (CARM1)/protein arginine Nmethyltransferase 4 (PRMT4) is identified as a binding partner of SRC-2/GRIP1 (glucocorticoid receptor-interacting protein 1) [71]. Recently, CARM1 shown to promote adipocyte differentiation by coactivating PPAR*γ* using cDNA microarray and serial analysis of gene expression (SAGE) [208]. CARM1 also stimulates transcriptional activation by nuclear receptors in combination with the p160 family of coactivators [71]. The p160/SRC coactivators recruit CBP/p300 and CARM1 via two activation domains, AD1 and AD2 [71]. AD1 binds CBP or p300, whereas AD2 has been shown to activate transcription through the recruitment of the arginine methyltransferase CARM1. The ternary complex of p160-CARM1-CBP/p300 functions synergistically to enhance transcriptional activation by nuclear receptors. CARM1 efficiently methylates three arginine residues (R714, R742 and R768) spanning aa 685–774 of CBP which are also conserved in p300 to transactivate SRC-2/GRIP-1 [209]. Other than these three methylated arginine residues, CARM1 also methylates KIX domain of CBP/p300 to block the interaction with KID domain of CREB (Cyclic AMP response element binding protein) [210]. Methylation is an irreversible process but peptidyl deiminase 4 removes methylated arginine from the p300 (Arg-2142) which is localized in the p160-binding domain to inhibit the bimolecular interactions between p300 and GRIP1. The functional significance of the methylation and demethylimination of the arginine residue of p300 may be a key mechanism in p300/CBP-p160-CARM1 coactivator synergy. Methylation of p300/CBP by CARM1 promotes a conformational change that allows the p300-p160 interaction in the complex and facilitates additional steps in transcriptional activation [211]. 

 Although CARM1 methylates CBP/p300 to enhance protein-protein interaction in the activated nuclear receptor complex, it also methylates histone tail, preferentially H3 which in turn relaxes chromatin to generate a docking site for coactivators and other transcriptional factors on the promoter of the target genes [71, 212, 213]. Methylation of the Q-rich domain of SRC-3 through CARM1 has an antagonizing activity on ER-mediated transcriptional activation [214]. During estrogen signaling methylation of SRC-3/AIB1 by CARM1 attenuates the transcriptional response by dissociating SRC-3/CARM1 coactivator complex from the ER receptor and thus completing a dynamic equilibrium of receptor-mediated coactivator assembly and disassembly at the promoter [214]. Mice deficient in CARM1 die at the perinatal stage emphasizing that CARM1 is crucial during late embryonic development or immediately after birth [215]. Methylation of CBP/p300 was shown to be abolished in the CARM1 knockout embryos and cells. Thus, it appears that CARM1 mediated methylation is needed for interaction between p/160 family of proteins and CBP/p300 to maintain general transcript integrity. 

## 15. Coactivator Activator (CoAA) with RNA Splicing Activity

The Coactivator activator (CoAA) was first identified as a protein associated with PRIP/TRBP (thyroid hormone receptor-binding protein) in a yeast two-hybrid screen [72]. CoAA functions as a general activator of transcription for several nuclear receptors and stimulates transcription through its interaction with the C-terminal of PRIP/TRBP [72]. CoAA interacts with both PRIP/TRBP and p300 in vitro. The PRIP-interaction domain on CoAA protein is localized at the central region, which is encoded by exon 2 of CoAA gene [72]. In addition, CoAA potently coactivates transcription mediated by multiple hormone-response elements and acts synergistically with PRIP/TRBP and CBP. Thus, CoAA appears to function as a coactivator associated protein. Apart from participating in PRIP/TRBPmediated transcription, CoAA also regulates alternative splicing in a promoter-dependent manner [72]. The N-terminal region of the CoAA protein contains two RNA recognition motifs (RRMs) at amino acid position 3–68 and 81–144. Both RRMs are composed of two conserved ribonucleoprotein (RNP) consensus motifs that regulate posttranslational RNA splicing [216]. Coactivator modulator (CoAM), a splice variant is generated as a result of alternative splicing of exon 2 of the CoAA. CoAM, which lacks a PRIP/TRBP-interacting domain, represses both PRIP/TRBP and CBP action suggesting that CoAM may modulate endogenous CoAA function [72]. In conclusion, CoAA and PIMT, both capable of interacting with PRIP/TRBP/NCoA6 appear to function as regulators of RNA processing. 

## 16. Conclusion

During the past 15 years, using yeast 2 hybrid screening [51, 52, 58, 59, 84], affinity pulldown of nuclear extracts via covalently bound ligand to the sepharose matrix [63], GST-receptor pulldown [64] and proteomic approaches [217], over 300 nuclear receptor transciption cofactors have been identified. Transcriptional control is a multistep process, a fact reflected in the diversity of the coregulators, and their intrinsic enzyme activities. These coregulators are possibly organized into stable, preformed multiprotein complexes, the modular character of which may facilitate the efficient assembly of functionally diverse complexes by a liganded nuclear receptor. In addition, the modular character of these complexes provides the potential for different activators to assemble diverse configurations of regulatory complexes at their cognate cis-acting elements. Emerging genomic and proteomic approaches promise to advance the characterization of coactivator proteins and their physiological functions. It should be worth noting that of the many cofactors, about 165 coactivators have been implicated to date in various human diseases [218]. Gene knockout mouse models have clearly established that Med1 is necessary for PPAR*α* and PPAR*γ* functions and that SRC-1, SRC-2, and SRC-3 are redundant for PPAR*α* function. It is anticipated that further studies of nuclear receptor coregulators and their complexes will yield significant insights into the basis of the complexity of signaling by PPARs and their ligands. 

## Figures and Tables

**Table 1:** Some known coactivator and coactivator associated proteins that regulate PPAR function.

Coactivator proteins	Enzyme activity	Function	References
SRC-1/NCoA-1	HAT	Histone acetylation	[51, 52]
SRC-2/TIF2/GRIP1	HAT	Histone acetylation	[53]
SRC3/pCIP/AIB1	HAT	Histone acetylation	[54, 55]
CBP/p300	HAT	Histone acetylation followed by recruitment of p160/SRCs	[56]
MED1/TRAP220/PBP	None	Anchor for Mediator complex	[57, 58]
PGC-1*α*	None	Recruit coactivator with HAT activities	[59]
PGC-1*β*/PERC	None	Recruit coactivator with HAT activities	[60]
PRIP/NCoA6	None	Recruit ASC complex	[61, 62]
PRIC285	Helicase	Chromatin remodeling by histone displacement and nucleosomal sliding	[63]
PRIC320/CHD9	Helicase	Chromatin remodeling by histone displacement and nucleosomal sliding	[64]
SWI/SNF	ATPase	ATP dependent mobilization of nucleosome	[65, 66]
BAF60a/SMARCD1	None	Recruit SWI/SNF complex	[67, 68]
BAF60c/SMARCD3	None	Recruit SWI/SNF complex	[69]

Coactivator-associated proteins			

PIMT/NCoA6IP	Methyltransferase	Methylation of caps of snRNAs and snoRNAs	[70]
CARM1/PRMT4	Methyltransferase	Potentiate SRCs by methylation of Histone H3	[71]
CoAA	None	RNA splicing	[72]
